# The effect of acute zonular occult outer retinopathy on retinal vessel oxygen saturation

**DOI:** 10.1007/s00417-026-07232-1

**Published:** 2026-04-10

**Authors:** Shahad Zaid Salim Al-Hamdani, Belal Al Tweish, Annekatrin Rickmann, Berthold Seitz, Hendrik PN Scholl, Nóra Szentmáry, Fabian N Fries, Maria della Volpe Waizel

**Affiliations:** 1 Department of Ophthalmology, University of Basel, Basel, Switzerland; 2 Sulzbach Eye Clinic, Knappschafts Hospital Sulzbach, Sulzbach, Saar Germany; 3 Department of Ophthalmology, Saarland University Medical Center, Homburg, Saar Germany; 4 Department of Clinical Pharmacology, Medical University of Vienna, Vienna, Austria; 5 Pallas Kliniken AG, Pallas Klinik Zürich, Zürich, Switzerland; 6 European Vision Institute, Basel, Switzerland; 7 Department of Experimental Ophthalmology, Saarland University, 66424 Homburg, Germany; 8 Department of Ophthalmology, Semmelweis-University, Mária u. 39, Budapest, 1085 Hungary

**Keywords:** acute zonular occult outer retinopathy, retinal oxygen metabolic function, inherited retinal diseases, retinal oximetry, retinitis pigmentosa

## Abstract

**Purpose:**

To measure the retinal vessel oxygen saturation alteration with retinal oximetry (RO) in patients with acute zonular occult outer retinopathy (AZOOR) and compare these findings with retinitis pigmentosa (RP) and controls.

**Methods:**

Prospective observational study examining 189 eyes of 98 patients: 12 eyes of 6 AZOOR patients (1♂5♀; 48.5 ± 19.2 years (mean ± SD) were compared to 77 eyes from 39 patients with RP (15♀ 24♂; 45.6 ± 14.7 years) and 100 eyes from 53 controls (31♀ 22♂; 40.2 ± 13.4 years). Main outcome measures were the mean arterial (A-SO_2_; %), venular (V-SO_2_; %) oxygen saturation, as well as their difference (A-V SO_2_; %) measured with the oxygen saturation tool of the Retinal Vessel Analyzer (IMEDOS Systems UG, Germany).

**Results:**

Eyes affected by AZOOR differed significantly from both RP and control eyes in terms of retinal oxygen metabolic parameters. Compared to controls, AZOOR eyes showed significantly higher A-SO₂ and V-SO₂ values (*p* = 0.031 and *p* = 0.044, respectively), while V-SO₂ was significantly lower in AZOOR compared to RP eyes (*p* = 0.035). A–V SO₂, which reflects retinal oxygen consumption, was significantly reduced only in RP eyes, but not in AZOOR eyes, when compared to controls.

**Conclusion:**

Assessments of retinal oxygen metabolism might contribute to a correct diagnosis and a better understanding of the pathogenesis of AZOOR and RP.

**Supplementary Information:**

The online version contains supplementary material available at https://doi.org/10.1007/s00417-026-07232-1.

## Key Messages

***What is known*:**

- Retinal oximetry (RO) is a useful tool for (differential) diagnosis of retinal diseases.
- Acute zonular occult outer retinopathy (AZOOR) often presents clinically as a phenocopy of retinitis pigmentosa (RP).

***What is new*:**

- RO function in AZOOR shows a different pattern when compared to RP, thus offering a fast and efficient way to find the correct clinical diagnosis.
- By offering a deeper understanding of retinal oxygen metabolism, RO may contribute to uncovering the underlying pathophysiological processes in AZOOR.

**Supplementary Information:**

The online version contains supplementary material available at https://doi.org/10.1007/s00417-026-07232-1.

## Introduction

Retinal oximetry (RO) is a medical Device that provides a non-invasive way to measure oxygen saturation levels in the retinal vessels by capturing the fundus [1]. It is well known that oxygen plays a crucial role in the maintenance of optimal visual function [2–3] and RO facilitates the assessment of in vivo retinal oxygen metabolic alterations [4–5].

Recent studies comparing the retinal vessel oxygen saturation in eyes affected by retinitis pigmentosa (RP) and other inherited retinal diseases (IRDs) with that of healthy controls have demonstrated significantly altered saturation levels in IRD patients [6–16]. These findings suggest that retinal oximetry (RO) could be a valuable tool for (differential) diagnosis and therapy monitoring [6, 7, 16], provided that influencing factors are carefully considered [17]. Consequently, RO offers a range of potential clinical applications, particularly for distinguishing phenocopies of IRDs.

Acute zonal occult outer retinopathy (AZOOR) is an inflammatory retinopathy within the wide range of white dot syndromes and is characterized by photoreceptor functional loss due to cell dysfunction [18–19]. Despite its distinct etiology, the clinical presentation of AZOOR often resembles that of an IRD, making it an important differential diagnosis, particularly in relation to RP [18–19]. AZOOR and RP share key features, including progressive visual field defects and outer retinal dysfunction detectable on electroretinography [18–22]. Both can eventually affect central and peripheral vision, and advanced stages may show similar outer retinal atrophy. AZOOR typically presents with a sudden or subacute onset of paracentral or peripheral field loss, often accompanied by photopsias, while early fundus findings can be subtle. Its course is usually monophasic or limited to a few episodes, with stabilization or partial recovery while RP develops insidiously, beginning with night blindness and slow peripheral field constriction, with characteristic fundus changes and continuous progression [6–16]. Currently neither etiology nor the pathology of the retinal pigment epithelium and photoreceptor epithelium complex malfunction caused by AZOOR is definitively understood [18–22]. Studies have suggested viral infections or an autoimmune etiology due to findings of antiretinal antibodies: Forooghian et al. evaluated 25 AZOOR patients for antiretinal antibodies for which all tested positive on Western blot analysis [21].

While RP and AZOOR are classified as distinct clinical entities, they both share an association with antiretinal antibodies, which are plausibly involved in the pathological changes of the outer retina [22]. Thus, analogous to RP [6–16], an alteration in oxygen metabolic function in patients with AZOOR is a reasonable assumption.

Therefore, this study aims to evaluate retinal vessel oxygen saturation in patients with AZOOR, in comparison to patients with RP and controls, in order to contribute to a better understanding of the underlying pathophysiology in AZOOR.

## Methods

This prospective observational double-armed study was performed on a total of 189 eyes of 98 patients. The first report presenting the effect of oximetry on choroideremia in a fourth arm of the study has already been published [16], the present data focusses on the analysis regarding the included patients of the AZOOR arm and includes a shared control group. All study participants were evaluated at a single ophthalmology center (Department of Ophthalmology, University of Basel, Switzerland) and were included between August 2020 and January 2025. The study received approval from the local ethics committee (Ethics Commission of Central and Northern Switzerland, EKNZ, Basel, Switzerland) and was authorized as a prospective observational study (trial number EKNZ BASEC 2020 − 00122).

Inclusion criteria for all patients and control subjects included Caucasian ethnicity and a refractive error with a spherical equivalent of less than ± 6 diopters for both myopia and hyperopia. Participants with ocular (e.g. cataract, diabetic retinopathy, glaucoma, any further retinal disease) or systemic conditions (e.g. hypertension, diabetes mellitus, cardiovascular diseases, respiratory disorders affecting oxygenation, anemia or polycythemia, and smoking) that could potentially affect retinal vessel oximetry measurements were excluded [17]. Additional exclusion criteria included poor-quality fundus oximetry images or a lack of willingness to participate.

All research procedures adhered to institutional guidelines and the principles of the Declaration of Helsinki. Written informed consent was obtained from all participants prior to examination. Each subject underwent a comprehensive ophthalmologic assessment, including refraction, best-corrected visual acuity (BCVA) using the ETDRS chart [23], slit-lamp examination, biomicroscopy, and fundoscopy.

All AZOOR and RP patients received comprehensive diagnostic evaluations tailored to their clinical presentation, performed by experienced, fellowship-trained retina specialists (M.d.V.W. and H.P.N.S.). These assessments included, among others, electrophysiological testing, short-wavelength fundus autofluorescence, optical coherence tomography (OCT), visual field testing using semi-automated kinetic perimetry (background illumination 10 cd/m²; V4e, III4e, I4e, III3e isopters assessed with the Octopus 900^®^), microperimetry, and genetic testing. Control subjects were healthy individuals with no evidence of ocular or systemic disease.

Prior to retinal oximetry measurements, both pupils were maximally dilated using Tropiphen eye drops (a preparation from our institutional pharmacy containing tropicamide 0.5% and phenylephrine 1%). Three drops were administered in each eye at 10-minute intervals.

### Retinal vessel oximetry acquisition

Retinal vessel oximetry was performed using a spectrophotometric oximetry system (IMEDOS Systems UG, Jena, Germany) in combination with a fundus camera (FF450, Carl Zeiss Meditec AG, Jena, Germany). Fundus images were captured using a DCC Digital Camera KY-F75 (JVC Inc., Yokohama, Japan) mounted on the Zeiss fundus camera, with a 50-degree field of view. The system’s software (VISUALIS; IMEDOS Systems UG) distinguishes oxygenated from deoxygenated hemoglobin by analyzing differences in light absorption at specific wavelengths, thereby determining the oxygen saturation in the retinal vessels being examined. RO imaging is conducted at two wavelengths: the green channel (548 ± 10 nm), which is relatively insensitive to oxygen, and the red channel (610 ± 10 nm), which is oxygen-sensitive [24–25].

A standardized imaging protocol centered on the optic disc was applied, utilizing two concentric rings in the peripapillary region: an inner ring with a radius of 1.0 optic disc diameters and an outer ring with a radius of 1.5 optic disc diameters. The area between these rings defined the region of interest for all measurements. For each eye, four test–retest fundus images were acquired [26]. Only images with optimal quality—defined as red channel illumination below 160 units and green channel illumination above 60 units on the respective scale—were included in the final analysis [27].

During RO analysis, all major arterioles and venules within the measurement zone were manually selected. The mean oxygen saturation in arterioles (A-SO₂) and venules (V-SO₂) were calculated, and their difference (A–V SO₂), representing an index of retinal oxygen metabolic consumption, was determined.

### Statistical analysis

The primary outcomes of the study included mean arterial oxygen saturation (A-SO₂; %), mean venous oxygen saturation (V-SO₂; %), the arteriovenous difference (A–V SO₂; %), as well as the average diameters of retinal arterioles (D-A; µm) and venules (D-V; µm). Oximetry measurements were compared across all three groups: AZOOR patients, RP patients, and controls.

Prior to statistical analysis, the normal distribution of all parameters was assessed using histograms and the Shapiro–Wilk test. To compare the subgroups, ANOVA with pairwise comparisons was conducted, and all reported p-values were adjusted using the Bonferroni correction. To rule out potential age-related differences between male and female participants within each group, unpaired t-tests were performed for all parameters.

ANOVA-based linear mixed-effects models were computed using SPSS^®^ (IBM SPSS Statistics^®^, Version 30.0.0.0 (172), including the Advanced Statistics package). This approach accounts for the inter-eye correlation in the same subject and is appropriate for analyzing repeated measurements. To assess the influence of subgroup membership on oximetry values, the model included eye (left or right), refractive spherical equivalent, and group as fixed factors, with the subject treated as a random factor. Results are reported as means and standard deviations (± SD) for each group, along with the corresponding p-values. A p-value of < 0.05 was considered statistically significant.

## Results

A total of 189 eyes of 98 patients were examined: 12 eyes of 6 AZOOR patients (1♂5♀; 48.5 ± 19.2 years (mean ± SD) were compared to 77 eyes from 39 patients with RP (15♀ 24♂; 45.6 ± 14.7 years) and 100 eyes from 53 controls (31♀ 22♂; 40.2 ± 13.4 years). BCVA was logMAR 0.61 ± 1.70 in AZOOR patients, logMAR 0.39 ± 0.67 in RP patients, and logMAR 0.00 ± 0.09 in controls. Both disease groups showed a significant functional deterioration compared to controls (*p* < 0.001). The clinical characteristics of all AZOOR patients are shown in Supplementary File 1.

### Oximetry results

I. Comparison of oxygen saturation values of diseased eyes to controls
  In controls, the mean ± SD for A-SO_2_ was 92.69 ± 5.95%, V-SO_2_ was 53.96 ± 9.25% and A-V SO_2_ was 38.73 ± 7.26%. In RP the corresponding values were: A-SO_2_ 98.73 ± 9.94%, V-SO_2_ was 62.06 ± 10.59% and A-V SO_2_ was 36.62 ± 8.85%; in AZOOR these measures were: A-SO_2_ 97.59 ± 8.01%, V-SO_2_ 54.30 ± 9.10% and A-V SO_2_ 43.30 ± 10.64%.
  In general, the disease subgroups differed from the controls when all oxygen metabolic parameters (A-SO₂, A–V SO₂, and V-SO₂) were considered: in RP, as previously reported, both A-SO₂ and V-SO₂ showed a statistically significant increase (*p* < 0.001), while A–V SO₂ exhibited a significant decrease (*p* < 0.001; Fig. 1). In contrast, while A-SO₂ and V-SO₂ were also significantly higher in AZOOR patients (*p* = 0.031 and *p* = 0.044, respectively), A–V SO₂ showed no significant difference compared to controls (*p* = 0.407; Fig. 1). The reported results in this section refer all to the comparison with controls.
  **Fig. 1:**
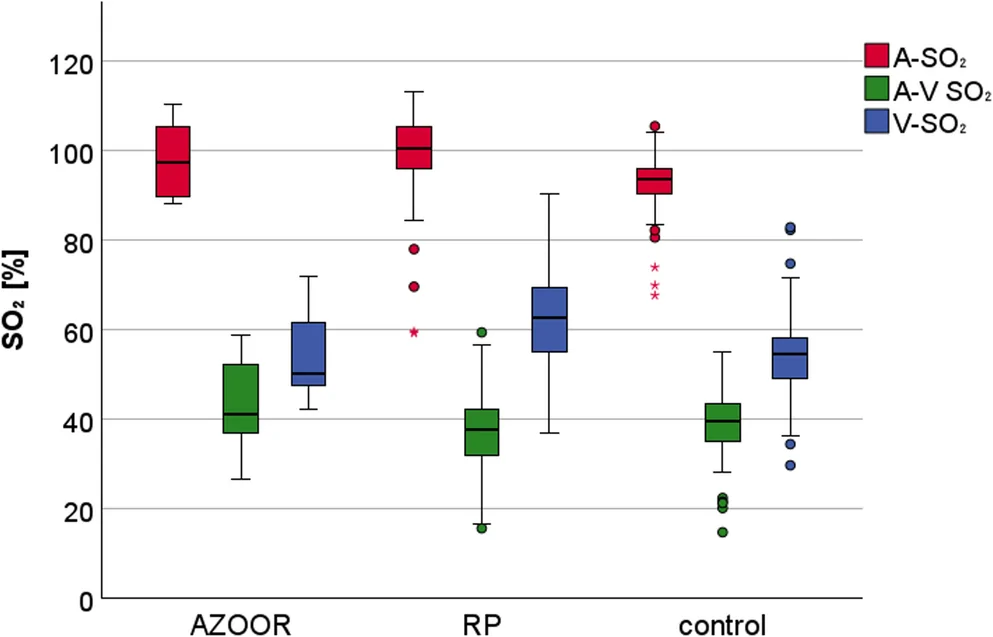
The box plot displays oxygen saturation levels in the retinal arterioles (red, A-SO₂), venules (blue, V-SO₂), and their difference (green, A–V SO₂). The y-axis represents vessel oxygen saturation percentages across the three groups shown on the x-axis (AZOOR, RP, control). The whiskers indicate the minimum and maximum values, while the box boundaries correspond to the 25th and 75th percentiles. The black horizontal line inside the box denotes the median. Outliers are depicted as individual circles
II. Comparison of oxygen saturation values of RP and AZOOR
  When comparing the diseased subgroups against each other, the AZOOR subgroup showed similar A-SO_2_ (*p* = 0.236) and A-V SO_2_ (*p* = 0.115) values as RP, however, V-SO_2_ was still significantly higher in RP than in AZOOR eyes (*p* = 0.035). The reported results in this section refer all to the comparison among the diseased eyes groups without comparing to controls.

I. Comparison of retinal vessel diameter values of diseased eyes to controls
  In controls, the mean ± SD for D-A was 92.85 ± 12.16 μm and for D-V 116.08 ± 15.40 μm. In RP the respective values were: for D-A 75.03 ± 12.36 μm and for D-V 94.46 ± 15.26 μm; in AZOOR these measures were: for D-A 86.02 ± 13.48 μm and for D-V 106.98 ± 15.57 μm.
  In general, the disease subgroups differed from the controls when all retinal vessel diameter parameters (D-A and D-V) were taken into account. In RP, as previously reported, both D-A and D-V showed a statistically significant narrowing of the retinal arterioles and venules (*p* < 0.001 for both comparisons; Fig. 2). Similarly, AZOOR eyes showed a significant narrowing of D-A and D-V compared to controls (*p* = 0.008 and *p* = 0.039, respectively; Fig. 2).
  **Fig. 2:**
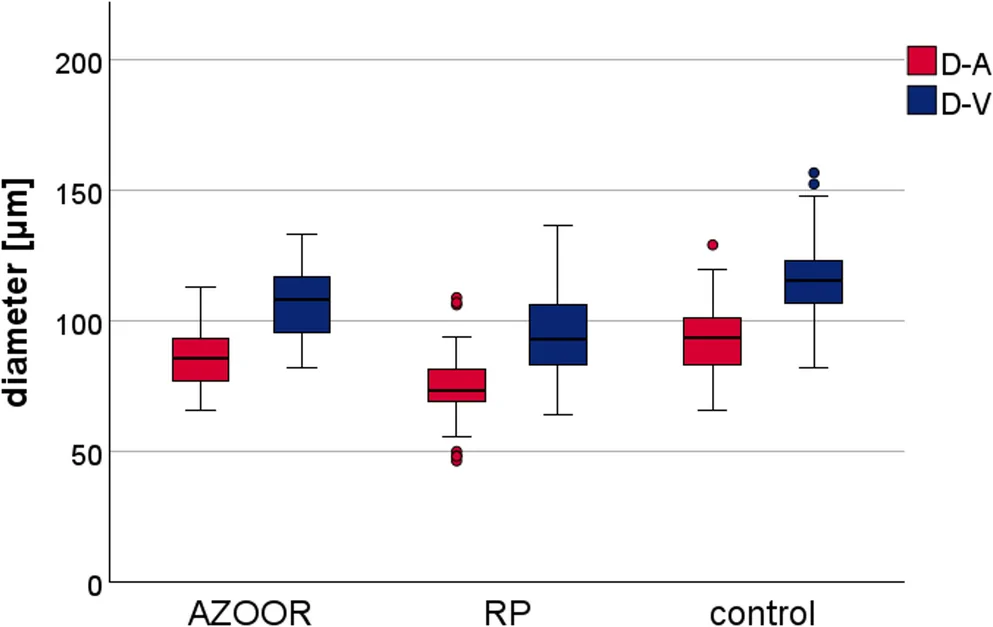
Box Plot analysis illustrates the retinal vessel diameters of the retinal arterioles (red, D-A) and venules (blue, D-V). The ordinate shows the diameters in micrometers for all three groups shown on the abscissa (AZOOR, RP, control). The upper and lower whiskers of the boxplot present the minimum and maximum value, the upper and lower borders of the box represent the 25th and 75th percentile respectively, and the black horizontal bar within the box represents the median value. Data points classified as outliers are marked as circles
II. Comparison of retinal vessel diameter values of RP and AZOOR
  When comparing the disease subgroups with each other, the RP subgroup showed significantly more pronounced narrowing of the retinal arterioles and venules compared to AZOOR, with both D-A and D-V being significantly lower (*p* = 0.014 and *p* = 0.028, respectively; Fig. 2).

## Discussion

Although AZOOR often presents with clinical features similar to those of RP [18–19], our findings reveal significant differences in retinal oxygen metabolism between the two conditions. The exact etiology of AZOOR, as well as the underlying mechanisms affecting the retinal pigment epithelium and photoreceptor complex, remain poorly understood [18–22].

Our study revealed significant alterations in retinal oxygen metabolism in AZOOR patients compared to controls, characterized by elevated arterial and venous hemoglobin oxygen saturation. Despite these changes, oxygen consumption in the AZOOR retina remained within normal limits. In contrast, when compared to the well-characterized IRD RP, different patterns emerged: while arterial oxygen saturation was comparable, RP patients showed significantly higher venous saturation and a marked reduction in oxygen consumption—the latter finding not observed in AZOOR.

Both local retinal oxidative stress and systemic factors appear to contribute to the apoptosis of cones, rods, and inner retinal cells in the pathological progression of RP [28–29]. Multiple studies have highlighted the critical role of oxygen metabolism in the development of RP showing elevated oxygen saturation in both retinal arterioles and venules, along with a decreased arterio-venous oxygen difference which represents the retinal oxygen consumption and is thought to result from photoreceptor cell loss [6–16]. Additionally, disruption of the blood-retina barrier allows protein-rich fluid and oxygen metabolites from the choriocapillaris to enter the extracellular space, exacerbating its hyperoxygenated state and intensifying oxidative damage [30–31]. These increased extracellular oxygen levels are believed to trigger vasoconstriction, leading to diminished retinal arterial blood flow and disturbed retinal hemodynamics [32]. This mechanism seems to be linked to a neurovascular remodeling [28, 30]: with progression of the dystrophy, the optic disc becomes pale and retinal vessels attenuate [30]. Our study confirms these alterations in retinal vascular oxygenation and the corresponding retinal vessel diameters in RP patients compared to controls.

The pathological changes in AZOOR are thought to be primarily affecting the outer retina, especially involving the photoreceptor layer and the retinal pigment epithelium (RPE). The inflammatory process is thought to target these outer retinal structures, leading to photoreceptor dysfunction and loss. Some studies also suggest involvement of the choroid: Hashimoto et al. assessed choroidal thickness in AZOOR-affected eyes and observed a progressive reduction at multiple locations—subfoveal, nasal, and temporal—over a 3 to 6-month period [33]. Mrejen et al. reported a rather sequential pattern of damage in AZOOR, involving the outer retina first, followed by the RPE and eventually the choroid. Their multimodal imaging approach, including SD-OCT, fundus autofluorescence (FAF), and indocyanine green angiography (ICGA), revealed that RPE and choroidal involvement occurs subsequent to photoreceptor disruption [34]. Our study seems to support the latter hypothesis: higher arterial and venous saturations appear to represent an impaired oxygen consumption in localized areas of the outer retinal photoreceptor layer where the inflammation seems to actually take place, however does not show an extensive pattern as found in RP. If the choroid was a primary focus, analogously to choroideremia [16], we would have found an opposite finding: a lower arterial and venous saturation. The insufficient supply of oxygen from the choroid would have sequentially contributed to the degeneration of photoreceptors and the RPE [16]. However, this seems not to be the case in AZOOR. First, there seem to be outer retinal disturbances that lead to oxygen metabolic dysfunction, followed by the choroidal thinning measured throughout the course of the disease [33].

Compared to controls, we found a significant attenuation of retinal arterioles and venules in AZOOR, however not as pronounced as in RP. The underlying mechanisms are thought to be mainly due to a decreased metabolic demand due to photoreceptor dysfunction or loss [33–35]. As AZOOR primarily affects the outer retina, especially photoreceptors, the retina’s oxygen and nutrient demand decreases with progression of the disease and, as a result, retinal blood flow is downregulated [33, 34]. The outer retinal hyperoxia observed in our study, likely resulting from reduced oxygen consumption, appears to contribute to vasoconstriction of both arterioles and venules. This mechanism resembles what has been described in RP. Additionally, in AZOOR, inflammation-related vascular dysregulation may further exacerbate vasoconstriction by impairing endothelial function and disrupting normal autoregulatory processes [33, 34]. Given the ongoing uncertainty surrounding the exact interplay of pathophysiological mechanisms in AZOOR [33–37], RO may become increasingly valuable in detecting early vascular and metabolic alterations in affected patients.

Our study has some limitations with a clinical heterogeneity of RP patients and a small sample size of the AZOOR subgroup. However, our previous work could show that oximetry changes happen early in the course of RP [8]. These significant changes can already be found in children affected by RP, even before neurovascular remodeling takes place [8]. Thus, oximetry values change gradually with more advanced stages, however statistically there is no significant difference between early and late RP stages [15].

Our findings provide the first RO data for AZOOR and demonstrate that RO may be a valuable modality for differentiating IRDs from phenocopies, including AZOOR. In conclusion, our findings provide deeper insight into the pathophysiology of AZOOR and its key differential diagnoses through the use of RO.

## Supplementary Information

Below is the link to the electronic supplementary material.

Supplementary Material 1. Clinical characteristics of all included patients with AZOOR are presented. (DOCX 3.00 MB)

## Data Availability

The original data are stored in the institutional database and can be provided on request.Trail registry: Ethikkommission Nordwest- und Zentralschweiz (EKNZ) Basel, SwitzerlandTrial registration number: EKNZ BASEC 2020-00122Date of registration: February 11, 2020.

## References

[1] Schweitzer D, Thamm E, Hammer M et al (2001) A new method for the measurement of oxygen saturation at the human ocular fundus. Int Ophthalmol 23:347–353 10.1023/a:1014458815482 11944861

[2] Stefánsson E, Wolbarsht ML, Landers MB (1983) In vivo O2 consumption in rhesus monkeys in light and dark. Exp Eye Res 37:251–256 10.1016/0014-4835(83)90159-8 6628573

[3] Wangsa-Wirawan ND, Linsenmeier RA (2003) Retinal oxygen: fundamental and clinical aspects. Arch Ophthalmol 121:547–557 10.1001/archopht.121.4.547 12695252

[4] Yu DY, Cringle SJ (2001) Oxygen distribution and consumption within the retina in vascularised and avascular retinas and in animal models of retinal disease. Prog Retin Eye Res 20:175–208 10.1016/s1350-9462(00)00027-6 11173251

[5] Yu DY, Cringle SJ (2005) Retinal degeneration and local oxygen metabolism. Exp Eye Res 80:745–751 10.1016/j.exer.2005.01.018 15939030

[6] Waizel M, Türksever C, Todorova MG (2018) The effect of autoimmune retinopathy on retinal vessel oxygen saturation. Eye (Lond) 32:1455–1462 10.1038/s41433-018-0122-3 PMC6137127 29786086

[7] Della Volpe-Waizel M, Zuche HC, Müller U et al (2020) Metabolic monitoring of transcorneal electrical stimulation in retinitis pigmentosa. Graefes Arch Clin Exp Ophthalmol 258:79–87 10.1007/s00417-019-04522-9 31713752

[8] Della Volpe Waizel M, Scholl HPN et al (2021) Retinal vessel oximetry in children with inherited retinal diseases. Acta Ophthalmol 99:52–60 10.1111/aos.14466 32573052

[9] Eysteinsson T, Hardarson SH, Bragason D et al (2014) Retinal vessel oxygen saturation and vessel diameter in retinitis pigmentosa. Acta Ophthalmol 92:449–453 10.1111/aos.12359 24767302

[10] Ueda-Consolvo T, Fuchizawa C, Otsuka M et al (2015) Analysis of retinal vessels in eyes with retinitis pigmentosa by retinal oximeter. Acta Ophthalmol 93:e446–450 10.1111/aos.12597 25403794

[11] Todorova MG, Scholl HPN, Della Volpe Waizel M (2021) The impact of macular edema on microvascular and metabolic alterations in retinitis pigmentosa. Graefes Arch Clin Exp Ophthalmol 259:643–652 10.1007/s00417-020-04913-3 32910309

[12] Zabek O, Camenzind Zuche H, Müller U et al (2021) Optical coherence tomography angiography findings in patients undergoing transcorneal electrical stimulation for treating retinitis pigmentosa. Graefes Arch Clin Exp Ophthalmol 259:1167–1177 10.1007/s00417-020-04963-7 PMC8102288 33037922

[13] Della Volpe Waizel M, Scholl HPN, Todorova MG (2021) Microvascular and metabolic alterations in retinitis pigmentosa and Stargardt disease. Acta Ophthalmol 99:e1396–e1404 10.1111/aos.14828 33973369

[14] Meral N, Zabek O, Camenzind Zuche H et al (2022) Metabolic Long-Term Monitoring of Transcorneal Electrical Stimulation in Retinitis Pigmentosa. Ophthalmic Res 65:52–59 10.1159/000519998 34607326

[15] Zabek O, Calzetti G, Prétot D et al (2022) Full-field sensitivity threshold and the relation to the oxygen metabolic retinal function in retinitis pigmentosa. Graefes Arch Clin Exp Ophthalmol 260:2517–2527 10.1007/s00417-022-05638-1 35355116

[16] Prétot D, Della Volpe Waizel M, Kaminska K et al (2025) Retinal oxygen metabolic function in choroideremia and retinitis pigmentosa. Graefes Arch Clin Exp Ophthalmol 263:379–385 10.1007/s00417-024-06659-8 PMC11868133 39394491

[17] Della Volpe Waizel M, Türksever C, Todorova MG (2020) Influence of cataract light scatters on retinal vessel oxygen saturation. Acta Ophthalmol 98:e56–e62 10.1111/aos.14200 31654499

[18] Gass JD (1993) Acute zonal occult outer retinopathy. Donders Lecture: The Netherlands Ophthalmological Society, Maastricht, Holland, June 19, 1992. J Clin Neuroophthalmol 13:79–97 8340485

[19] Gass JD, Agarwal A, Scott I (2002) Acute Zonal Occult Outer Retinopathy: A Long-term Follow-up Study. Am J Ophthalmol 134:329–339 10.1016/s0002-9394(02)01640-9 12208243

[20] Gass JD (2003) Are acute zonal occult outer retinopathy and the white spot syndromes (AZOOR complex) specific autoimmune diseases? Am J Ophthalmol 135:380–381 10.1016/s0002-9394(03)00030-8 12614758

[21] Forooghian F (2017) Prevalence of Antiretinal Antibodies in Acute Zonal Occult Outer Retinopathy: A Comprehensive Review of 25 Cases. Am J Ophthalmol 179:210–211 10.1016/j.ajo.2017.01.036 28522028

[22] Bird A, Holder G (2016) Acute Zonal Occult Outer Retinopathy. In: Zierhut M, Pavesio C, Ohno S, Oréfice F, Rao NA (eds) Intraocular Inflammation. Springer-, Berlin Heidelberg, pp 909–915

[23] Flynn HW Jr, Chew EY, Simons BD et al (1992) Pars plana vitrectomy in the Early Treatment Diabetic Retinopathy Study. ETDRS report number 17. The Early Treatment Diabetic Retinopathy Study Research Group. Ophthalmology 99:1351–1357 10.1016/s0161-6420(92)31779-8 1407968

[24] Hammer M, Thamm E, Schweitzer D (2002) A simple algorithm for in vivo ocular fundus oximetry compensating for non-haemoglobin absorption and scattering. Phys Med Biol 47:233–238 10.1088/0031-9155/47/17/403 12361226

[25] Hammer M, Vilser W, Riemer T et al (2009) Diabetic patients with retinopathy show increased retinal venous oxygen saturation. Graefes Arch Clin Exp Ophthalmol 247:1025–1030 10.1007/s00417-009-1078-6 19404666

[26] Türksever C, Valmaggia C, Orgül S et al (2014) Retinal vessel oxygen saturation and its correlation with structural changes in retinitis pigmentosa. Acta Ophthalmol 92:454–460 10.1111/aos.12379 24767408

[27] Türksever C, Orgül S, Todorova MG (2015) Reproducibility of retinal oximetry measurements in healthy and diseased retinas. Acta Ophthalmol 93:e439–e445 10.1111/aos.12598 25430037

[28] Cottet S, Schorderet DF (2009) Mechanisms of apoptosis in retinitis pigmentosa. Curr Mol Med 9:375–383 10.2174/156652409787847155 19355918

[29] Shen J, Yang X, Dong A et al (2005) Oxidative damage is a potential cause of cone cell death in retinitis pigmentosa. J Cell Physiol 203:457–464 10.1002/jcp.20346 15744744

[30] Marc RE, Jones BW (2003) Retinal remodeling in inherited photoreceptor degenerations. Mol Neurobiol 28:139–147 10.1385/MN:28:2:139 14576452

[31] Milam AH, Li ZY, Fariss RN (1998) Histopathology of the human retina in retinitis pigmentosa. Prog Retin Eye Res 17:175–205 10.1016/s1350-9462(97)00012-8 9695792

[32] Beutelspacher SC, Serbecic N, Barash H et al (2011) Retinal blood flow velocity measured by retinal function imaging in retinitis pigmentosa. Graefes Arch Clin Exp Ophthalmol 249:1855–1858 10.1007/s00417-011-1757-y 21874346

[33] Hashimoto Y, Saito W, Saito M et al (2017) Relationship between Choroidal Thickness and Visual Field Impairment in Acute Zonal Occult Outer Retinopathy. J Ophthalmol 2017: 2371032 10.1155/2017/2371032 PMC5516752 28758030

[34] Mrejen S, Khan S, Gallego-Pinazo R et al (2014) Acute Zonal Occult Outer Retinopathy: A Classification Based on Multimodal Imaging. JAMA Ophthalmol 132:1089–1098 10.1001/jamaophthalmol.2014.1683 24945598

[35] Iuliano M, Lombardo M, Falsini B et al (2025) Structural, Functional, and Cellular Analysis of a Case of Acute Zonal Occult Outer Retinopathy (AZOOR). Biomedicines 13:1521 10.3390/biomedicines13071521 PMC12292167 40722597

[36] Roy R, Dutta Majumder P (2024) Current understanding of acute zonal occult outer retinopathy (AZOOR). Indian J Ophthalmol 72:935–937 10.4103/IJO.IJO_3228_23 PMC11329826 38454854

[37] Cicinelli MV, Ramtohul P, Marchese A et al (2023) Latest advances in white spot syndromes: New findings and interpretations. Prog Retin Eye Res 97:101207 10.1016/j.preteyeres.2023.101207 37574123

