# Supplementary material for: The effect of acute zonular occult outer retinopathy on retinal vessel oxygen saturation
Source: Graefes Arch Clin Exp Ophthalmol. 2026 Apr 10;264(10):3123–9. doi: 10.1007/s00417-026-07232-1 (PMC13653951; doi:10.1007/s00417-026-07232-1)
Supplement: Supplementary file 1 [file 417_2026_7232_MOESM1_ESM.docx]

***Patient#1***

A 22-year-old so far healthy lady presented with flickering and a slightly increased light sensitivity on both eyes. Ophthalmological examination revealed a best corrected visual acuity (BCVA, Snellen charts) of 20/25, as well as normal anterior segment findings on both eyes. The funduscopy presented with slight pigmentary changes of the posterior pole. Multimodal retinal imaging revealed a preserved subfoveal IS/OS junction line with parafoveal discontinuation (macular OCT, supplemental Fig. S1a). Fundus autofluorescence (FAF) showed a trizonal defect appearance with normal FAF outside of a speckled hyperautofluorescence around the macula and central areas of atrophy with hypoautofluorescence at the posterior pole (supplemental Fig. S1b). The visual field measured with semi-automated kinetic perimetry (V4e (blue), III4e (red), I4e (green), III3e (pink) isopters, Octopus 900®, Haag-Streit AG Bern, Switzerland) was within normal outer limits on both eyes (supplemental Fig. S1c), however, a more detailed examination with microperimetry (MP3, Nidek Co, Japan) revealed a stable fixation on both eyes and a reduced sensitivity in regions corresponding to the atrophic zones as found in FAF (supplemental Fig. S1d). Full-field electroretinography (ffERG, Diagnosys LLC Espion system; ISCEV standard) presented normal scotopic, photopic and 30 Hz flicker respnses (supplemental Fig. S1e). Taking all of the results together, we suspected an Acute Zonal Occult Outer Retinopathy (AZOOR).

***Patient#2***

A 36-year-old lady was referred by her private ophthalmologist with a long history of flickering phenomena and visual deterioration of her right eye since the age of 16y. The visual impairment persisted and remained stable ever since. Various extensive medical, neurological, ophthalmological and serological tests had already excluded HLA-A29 positivity, tuberculosis, syphilis, Human Immunodeficiency Virus (HIV), borrelia, human cytomegalovirus (CMV), Epstein–Barr virus (EBV), bartonella, herpes simplex virus, toxoplasmosis, yersinia and listeria, furthermore anti-nuclear antibody (ANA), Angiotensin Converting Enzyme (ACE), erythrocyte sedimentation rate (ESR) and C-reactive protein (CRP) were within the normal range. Our ophthalmological examination revealed a decreased BCVA on her right eye (20/32), the left eye was 20/20. Anterior segments presented with normal findings. The funduscopy showed a peripapillary diffuse retinal atrophy on the right eye with central and peripheral pigment epithelial changes, the left eye presented with slight pigmetary changes at the posterior pole. FAF of the right eye showed a trizonal defect appearance with a peripapillary lesion with speckled hyperautofluorescence and central areas of atrophy with hypoautofluorescence around the optic disc (supplemental Fig. S2b), also the left eye presented with hypoautofluorescent atrophic areas in the macula and a hyperautofluorescent bleaching effect after the OCT examination. Corresponding to the lesion found in the FAF, the macular OCT presented a discontinued IS/OS junction line and atrophy signs of the outer retina on both eyes (supplemental Fig. S2a). The full-field ERG showed extinguished scotopic, photopic and 30 Hz responses on the right eye with preserved responses on the left eye (supplemental Fig. S2e). The white full-field stimulus threshold (FST) was reduced on both eyes: -62 dB on the right eye and -68 dB on the left eye. Visual field examination (semi-automated kinetic perimetry, V4e, III4e, I4e, III3e isopters, Octopus 900®, Haag-Streit AG Bern, Switzerland) showed a restriction of the nasal and superior outer areas on the right eye and normal values on the left eye, while a more detailed examination with microperimetry (MP3, Nidek Co, Japan) revealed a stable fixation on both eyes, however a reduced sensitivity in regions corresponding to the atrophic zones as found in FAF and OCT (supplemental Fig. S2c and S2d). Taking all of the results together, we suspected AZOOR with more pronounced changes on the right than the left eye.

***Patient#3***

A 75-year-old lady visited our consultation hour for a second opinion with a long history of flickering phenomena and visual deterioration of both eyes since >20 years. Her BCVA had dropped to 20/50 on her right eye and nulla lux on her left eye. Ophthalmological examination revealed normal anterior segment findings with pseudophakia. The funduscopy showed retinal vascular attenuation, a macula with beaten bronze appearance and bone spicule-like RPE changes in the mid periphery on both eyes (supplemental Fig. S3d). The macular OCT revealed a discontinued IS/OS junction line with a small subfoveal area of preserved outer segments, the left eye further showed an epiretinal membrane (supplemental Fig. S3a). Visual field examination (Octopus® perimetry, program: Low Vision, 30°) showed a concentric peripheral loss on both eyes (supplemental Fig. S3c) with corresponding hypofluorescent areas of atrophy with a “marmorata” aspect along the vascular arcades in the FAF images (supplemental Fig. S3b). A multifocal electroretinography (mfERG) had already been performed externally which showed a strong signal reduction corresponding to the atrophic areas seen in the OCT and FAF imaging. The patient did not wish for another repetition of the eletrophysiological measurements in our center. Furthermore, as the patient had already been thoroughly checked by her previous Ophthalmology department including medical and neurological examinations we confirmed the diagnosis of a long-standing AZOOR on both eyes.

***Patient#4***

A 62-year-old gentleman presented himself in our consultation hour for a second opinion with a two years history of visual deterioration on both eyes, visual field restrictions around the blind spot and the suspected diagnosis of an “atypical” retinitis pigmentosa. His BCVA was 20/25 on his right eye and 20/40 on his left eye. Ophthalmological examination showed normal anterior segment findings with a beginning cataract. The funduscopy revealed normal retinal vessels, however a macula with pigment epithelial changes and bone spicule-like RPE changes in the mid periphery on both eyes. The macular OCT presented with a discontinued IS/OS junction line with a small subfoveal area of preserved outer segments (supplemental Fig. S4a). Visual field examination (Octopus® perimetry, program: G, 30°) showed an absolute scotoma around the blind spot with extentions into the center on both eyes (supplemental Fig. S4c) which corresponded with the FAF imaging that presented a trizonal defect appearance with normal FAF outside of a speckled hyperautofluorescence surrounding the macula and the optic nerve and areas of atrophy with hypoautofluorescence within the lesion (supplemental Fig. S4b). Microperimetry (MP3, Nidek Co, Japan) revealed a stable fixation on both eyes, however a reduced sensitivity in regions corresponding with the atrophic zones as found in FAF and OCT (supplemental Fig. S4d). The ffERG showed preserved scotopic, photopic and 30 Hz flicker responses, however with amplitudes in the lower normal range (supplemental Fig. S4e). Taking all findings together we suspected AZOOR on both eyes.

***Patient#5***

A 34-year-old lady diagnosed with AZOOR since 2 years visited our center for the study participation. She reported about a first epidode three years ago with flickering and visual field disturbances beginning on her left eye that started few days after a short period with gastroeneteritis of unknown etiology. Ophthalmological examination revealed her BCVA to be 20/20, as well as normal anterior segment findings on both eyes. The funduscopy presented with slight pigmentary changes of the posterior pole in both eyes and peripheral pigment epithelial changes in the left eye (supplemental Fig. S5d). Fundus autofluorescence (FAF) showed a trizonal defect on the right eye with normal FAF outside of a speckled hyperautofluorescence around the optic disc and peripapillary areas of atrophy with hypoautofluorescence. The left eye presented with a speckled hyperautofluorescence of the macula and hypoautofluorescent mottling outside the vascular arcades (supplemental Fig. S5b). Multimodal retinal imaging revealed a centrally preserved IS/OS junction line with mid-peripheral discontinuation that correlated with the lesions seen in the FAF (macular OCT, supplemental Fig. S5a). Visual field examination (Carl Zeiss Humphrey Field Analyzer® perimetry, program: 30°) showed a concentric restriction on the left eye and a scotoma around the blind spot on the right eye (supplemental Fig. S5c). ffERG presented normal scotopic, photopic and 30 Hz flicker responses on the right eye and extinguished scotopic and reduced photopic and 30 Hz flicker responses on the left eye (supplemental Fig. S5e). Taking all of the results together, we confirmed the diagnosis of AZOOR.

***Patient#6***

A 55-year-old lady was referred to our center with a six moths history of visual field restrictions and flickering on her left eye which occurred after a short episode of flu symptoms. Her BCVA was 20/20 on both eyes. Ophthalmological examination showed normal anterior segment findings with a clear lens. The funduscopy revealed normal retinal vessels, however a macula with pigment epithelial changes on both eyes. The macular OCT presented with a discontinued IS/OS junction line with a subfoveal area of preserved outer segments (supplemental Fig. S6a). The visual field measured with semi-automated kinetic perimetry (V4e, III4e, I4e, III3e isopters, Octopus 900®, Haag-Streit AG Bern, Switzerland) was within normal outer limits on both eyes (supplemental Fig. S6c), however, a more detailed examination with static perimetry (Octopus®, program: N, 60°) revealed scotomas around the blind spot on both eyes with extension to center on the left eye (supplemental Fig. S6d). FAF imaging of the left eye presented a trizonal defect appearance with normal FAF outside of a speckled hyperautofluorescence surrounding the macula and the optic nerve and areas of atrophy with hypoautofluorescence within the lesion. The right eye showed similar findings, however less pronounced (supplemental Fig. S6b). The ffERG showed preserved scotopic, photopic and 30 Hz flicker responses, however with amplitudes in the lower normal range on the left eye (supplemental Fig. S6e). Taking all findings together we suspected AZOOR on both eyes.

Supplemental Figure S1: presented are the clinical findings of AZOOR patient #1.

Supplemental Figure S2: presented are the clinical findings of AZOOR patient #2.

Supplemental Figure S3: presented are the clinical findings of AZOOR patient #3.

Supplemental Figure S4: presented are the clinical findings of AZOOR patient #4.

Supplemental Figure S5: presented are the clinical findings of AZOOR patient #5.

Supplemental Figure S6: presented are the clinical findings of AZOOR patient #6.
